# Genome Sequence of “*Candidatus* Thioglobus autotrophica” Strain EF1, a Chemoautotroph from the SUP05 Clade of Marine Gammaproteobacteria

**DOI:** 10.1128/genomeA.01156-15

**Published:** 2015-10-22

**Authors:** Vega Shah, Robert M. Morris

**Affiliations:** School of Oceanography, University of Washington, Seattle, Washington, USA

## Abstract

Chemoautotrophic marine bacteria from the SUP05 clade of marine gammaproteobacteria often dominate low-oxygen waters in upwelling regions, fjords, and hydrothermal systems. Here, we announce the complete genome sequence of “*Candidatus* Thioglobus autotrophica” strain EF1, the first cultured chemoautotrophic representative from the SUP05 clade.

## GENOME ANNOUNCEMENT

Chemoautotrophic members of the SUP05 clade of marine gammaproteobacteria are abundant in the suboxic ocean (1–4). They are of particular interest because of their potential to mediate biogeochemical cycles in anoxic fjords, upwelling zones, and sulfidic regions, like the Black Sea (5–7). Cultivation-independent studies suggest that SUP05 organisms have critical roles in carbon fixation, denitrification, and sulfur oxidation (5, 8, 9). Here, we announce the complete genome sequence of “*Candidatus* Thioglobus autotrophica” strain EF1, the first cultured chemoautotrophic representative from the SUP05 clade. “*Ca.* Thioglobus autotrophica” EF1 is a member of the *sup05* subclade (designated here by lowercase italics) and was isolated from a redox gradient (60 m) in Effingham Inlet, British Columbia, Canada. The complete genome of “*Ca.* Thioglobus autotrophica” EF1 is circular, 1,512,449 bp long, and codes for 1,637 genes.

Genomic DNA was extracted from a total of 62 pure cultures grown anaerobically in 100-ml bottles. Cells were grown to early stationary phase (~2.0 × 10^6^ cells/ml) and then collected on sterile Supor-200 0.2-µM polyethersulfone filters (Pall Corporation, Port Washington, NY). DNA was extracted as previously described (10). Clone library preparation for genome sequencing was performed at the University of Washington’s Genome Science Department using Pacific Bioscience’s single-molecule real-time (SMRT) sequencing technology. *De novo* assembly of the “*Ca.* Thioglobus autotrophica” EF1 genome was conducted using Hierarchical Genome Assembly Process (HGAP), as previously described (11). Briefly, single reads were mapped to seed reads, a Celera assembler created overlapping consensus sequences, and the remaining indel and base substitution errors were removed. This method has been found to produce highly accurate and complete *de novo* assemblies for small prokaryotic genomes (12). HGAP assembly of the “*Ca.* Thioglobus autotrophica” EF1 genome resulted in a single contiguous sequence that was closed using a single PCR. The complete genome sequence of “*Ca.* Thioglobus autotrophica” EF1 used 100% of the cleaned reads, with an average coverage of 106×, indicating high confidence in a single circular chromosome of 1,512,449 bp in length. Protein-coding sequences were identified and annotated via the NCBI Prokaryotic Genome Annotation Pipeline and were checked against RAST annotations (13, 14), IMG annotations (15), and, in some cases, by phylogenetic analyses. Discrepancies were corrected, and final annotations were submitted to NCBI.

### Nucleotide sequence accession number.

The complete genome sequence of “*Ca.* Thioglobus autotrophica” strain EF1 is available in GenBank under the accession no. CP010552.
